# A Novel Fault Detection and Identification Framework for Rotating Machinery Using Residual Current Spectrum

**DOI:** 10.3390/s21175865

**Published:** 2021-08-31

**Authors:** Widagdo Purbowaskito, Chen-Yang Lan, Kenny Fuh

**Affiliations:** 1 Department of Mechanical Engineering, National Taiwan University of Science and Technology, Taipei 10607, Taiwan; widagdo.purbowaskito@uajy.ac.id; 2 Department of Industrial Engineering, Universitas Atma Jaya Yogyakarta, Yogyakarta 55281, Indonesia; 3 Jhunan Facility Engineering Division Group, Innolux Corporation, Jhunan Science Park, Zhunan 35053, Taiwan; kenny.fuh@innolux.com

**Keywords:** model-based fault detection, fault identification, induction motors, residual spectrum, subspace identification, Q-function

## Abstract

A novel framework of model-based fault detection and identification (MFDI) for induction motor (IM)-driven rotating machinery (RM) is proposed in this study. A data-driven subspace identification (SID) algorithm is employed to obtain the IM state-space model from the voltage and current signals in a quasi-steady-state condition. This study aims to improve the frequency–domain fault detection and identification (FDI) by replacing the current signal with a residual signal where a thresholding method is applied to the residual signal. Through the residual spectrum and threshold comparison, a binary decision is made to find fault signatures in the spectrum. The statistical *Q*-function is used to generate the fault frequency band to distinguish between the fault signature and the noise signature. The experiment in this study is performed on a wastewater pump in an existing industrial facility to verify the proposed FDI. Two faulty conditions with mathematically known and mathematically unknown faulty signatures are experimented with and diagnosed. The study results present that the residual spectrum demonstrated to be more sensitive to fault signatures compare to the current spectrum. The proposed FDI has successfully shown to identify the fault signatures even for the mathematically unknown faulty signatures.

## 1. Introduction

Primarily, the industrial RM utilizes an IM as the actuator, such as a pump, fan, conveyor, or compressor. In the factory, IM-driven RM often run not in a proper condition in which their efficiency may reduce, and the energy cost may increase. If this condition stays for a more extended period, it will lead to machine failure and may stop the entire production process with the risks of safety, expensive production loss, a time-consuming, and costly repairing process. As the industrial systems become more complex and expensive, the demand to improve the reliability and safety of the systems subjected and exposed to faults and failures such as the IM-driven RM is increasing. The development of IM-driven RM condition monitoring and FDI has a promising contribution in reducing the maintenance cost and extending the operation time.

Popular and promising FDI strategies in recent years include motor current signature analysis (MCSA) and vibration analysis [1,2,3]. Both methods examine the abnormal harmonic modulation at the specific fault characteristic frequencies in the frequency spectrum by utilizing the Fast Fourier Transform (FFT) algorithm. Nevertheless, the current signal provides better information than the vibration signal under different operation conditions [4]. The availability of a low-cost step-down current transformer sensor and a high sampling-frequency resolution data-acquisition (DAQ) card also provides cheap and reliable measurement of the IM current signal in industrial plants. The current signal can be measured from the IM control panel, far away from the RM itself. Thus, it makes the current measurement safer than the vibration. As MCSA examines the specific fault characteristic frequencies, this method can effectively isolate the faults. However, MCSA fundamentally relies on a minimal magnitude fault frequency vulnerable to harsh industrial noise and input voltage quality interference. Thus, the detection and identification results can easily be distorted, and it reduces the effectiveness of MCSA based FDI.

A novel framework is proposed to improve the effectiveness of frequency-domain fault identification against noise and input voltage quality interference. The improvement relies on the utilization of the residual current signal instead of the current signal in the frequency-domain analysis. The proposed method considers the relationship between the input voltage and the output current through the identified IM model. The residual signal is obtained from the subtraction of the measured output current and the estimated current signal from the identified IM model. Thus, this method is also called MFDI. MFDI is an FDI approach based on the analytical redundancy in which a mathematical model of the system is utilized with some estimation/identification techniques to generate the estimated system output and obtaining the residual of the system output [5]. Generally, the MFDI process consists of system identification/parameter estimation, residual generation, and residual evaluation [5,6]. MFDI relies on the accuracy of the system model description so that the fault indication can be achieved from the residual of the system output. The system model itself can be obtained through the derivation of the first principle and data-driven identification [7,8].

Recently, model derivations have been proposed to perform an MFDI approach on the IM, such as modification of the IM model with stator-faults and broken rotor bars [9,10,11,12], current estimator model using first and third harmonic magnitudes and phase angles of the stator current [13], and cascade modeling of IM and centrifugal pump with structural analysis derivation [14]. However, it is essential to note that the first principle derivation of the IM model,

1. Considers its coupled mechanical or driven system [14];
2. Requires prior knowledge regarding the specific operation conditions [13];
3. Can be time-consuming, nontrivial, and inaccurate due to the dynamic assumptions, and some of the model uncertainties are not quantifiable [15].

Thus, to overcome these, a data-driven model identification approach is considered to obtain the IM model that describes the actual condition of the IM. The data-driven model identification will be accomplished by applying the black-box identification based on SID.

Since it is data-driven, the IM model will be identified based on the measured input-output voltage and current data at a specific particular time. Therefore, the IM model is considered as a linear quasi-steady-state discrete state-space model. SID algorithm has been applied in the model identification for MFDI method in different cases and provides robust residual generator [6,8,16,17,18]. SID algorithm estimates the state sequences directly from the measured input–output data by using the orthogonal and the oblique projections of some particular data block Hankel matrix row spaces into another particular data block Hankel matrix row spaces. The projections are executed after the QR decomposition is applied to the block Hankel matrices. Then, the Singular Value Decomposition (SVD) is used to determine the system order, the observability matrix, and the state sequences [19]. Unlike the derivation of the first principle, SID-based model identification provides a general, and straightforward parameterization of a multivariable system [20]. However, the model parameters do not have physical meanings. Thus, changes and variations in these parameters cannot be used to understand the cause-effect in FDI. Although the parameter’s physical meaning may be lost, this identified model provides an adequately accurate dynamic representation of the IM behavior at a specific time as it is not derived under any dynamic assumptions. The significant advantage of the SID algorithm is non-iterative with no nonlinear optimization involved so that there is a guarantee of convergence of the objective criterion [21]. This advantage is well-suited for industrial practices as it does not require a long computational time.

FDI is a challenging issue, particularly in a low signal-to-noise ratio (SNR) condition such as in an actual industrial plant. A frequency-domain diagnosis framework must be able to provide reliable performance in evaluating the faulty condition. The proposed MFDI provides novelties to overcome those particular challenges. It employs a data-driven IM model identification for fast computational time and accurate input voltage and outputs current relationship description. The IM model filters the dominant input voltage harmonics and noises from the output current spectrum and presents it as the residual spectrum. A threshold as a benchmark for evaluating the residual spectrum is generated to identify the fault signatures.

**Figure 1:**
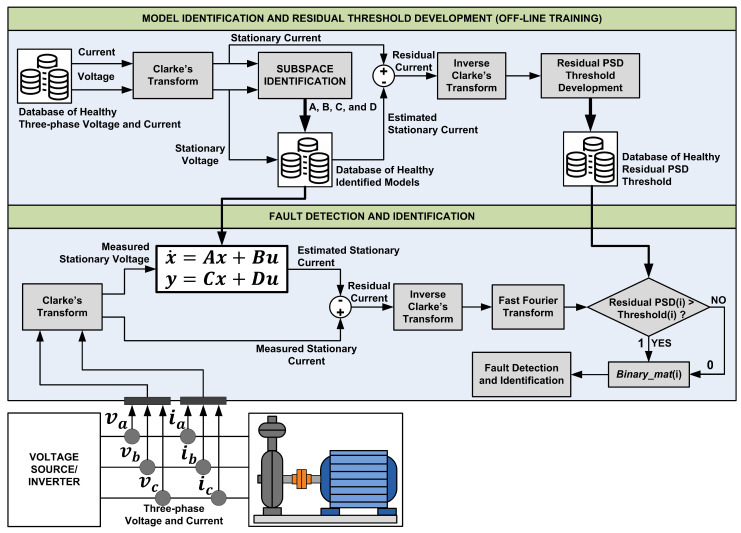
Proposed model-based fault detection and identification framework.

The general framework of the proposed MFDI is shown in Figure 1. The experiment in this study has been conducted in an industrial wastewater centrifugal pump driven by an IM with a flexible coupling transmission. Five sections are presented in this paper. Section 1 explains the background of study and motivation. Section 2 provides the motor current signal and its frequency spectrum pattern. Section 3 describes the data-driven-based model identification, while Section 4 provides details of the FDI algorithm. Section 5 presents the experimental setups. Section 6 presents the experimental results and their discussions, and Section 7 the conclusions and the future works of this study are presented.

## 2. Motor Current Signature

### 2.1. Motor Current Signal Description

The motor current signal reflects the electrical relationship between the stator and the rotor, where helpful information regarding the IM condition can be observed in its frequency spectrum. For instance, the presence of electrical faults such as stator short circuits and broken rotor bar; and mechanical faults such as bearing faults, misalignment, air-gap eccentricity, and mechanical looseness induces additional frequency components in the motor current stator signal [22,23,24,25,26]. Besides, the inherent defect in the IM design or construction and the driven-systems variation may induce multiple harmonics into the motor current signal. While in actual industrial operations, the motor current frequency spectrum is vulnerable to background noise distortion and voltage harmonics interference. Therefore, the theoretical motor current signal in faulty condition can be decomposed as follows,

(1) $$I\left(t\right)=\sum_{i=1}^{K}A_{i}\cos\left(2\pi f_{i}t+\phi_{i}\right)+n\left(t\right),$$

where $n\left(t\right)∼N\left(0,\sigma^{2}\right)$ denotes the Gaussian white noise coming from the driving voltage signal and measurement noises, $f_{i}$ is the frequency component, $A_{i}$ is the amplitude, and $\phi_{i}$ is the initial phase. It is important to note that in Equation (1), the motor current harmonic and fault characteristic frequency are not described separately as in some cases, the fault characteristic frequency overlaps the harmonics [27,28].

The main idea of MFDI is to generate an output estimate of a system and obtain the residual output. The motor current signal is estimated using the motor model obtained from the input-output system identification in this study. Theoretically, while keeping the voltage harmonic and driving voltage noise, the estimated motor current signal is free from fault and measurement noise components. Therefore, subtracting the estimated current signal from the measured current signal can exclusively isolate the residual harmonic in which its magnitude is much smaller than in the current harmonic, fault frequency components, and measurement noise components.

(2) $$r\left(t\right)=I\left(t\right)-\hat{I}\left(t\right)$$

(3) $$r\left(t\right)=\sum_{i=1}^{K}r_{i}\cos\left(2\pi f_{i}^{r}t+\theta_{i}\right),$$

where $\hat{I}\left(t\right)$ is the estimated current signal, r(t) is the residual current signal, $r_{i}$ is the residual signal amplitudes, $f_{i}^{r}$ is the residual frequency, and *i* is an integer value correspond to the residual frequency index with *K* indexes length. Now, the so-called residual frequency $f_{i}^{r}$ corresponds to residual harmonic, fault, and measurement noise frequency components where their presence may overlap each other. The frequency spectrum of Equations (1) and (3) can be obtained by applying the FFT algorithm where it is based on this following Discrete Fourier Transform (DFT) [29],

(4) $$\begin{array}{c} X\left(k\right)=\sum_{n=0}^{N-1}x\left(n\right)e^{-j\frac{2\pi }{N}kn}\\ X\left(k\right)=\sum_{n=0}^{N-1}x\left(n\right)\left(\cos\left(\frac{2\pi }{N}kn\right)-j\sin\left(\frac{2\pi }{N}kn\right)\right), \end{array}$$

where k=0,1,2,3,⋯,N−1, and *N* is the length of data.

### 2.2. Fault Characteristic Frequency

The development of MCSA recognizes the additional frequency components induced by the faulty conditions as the fault characteristic frequencies, as they are described in Equation (1). The proposed FDI method isolates fault characteristic frequencies as a frequency pattern or band in which it is treated as a subset frequency for detection and identification evaluation. Generally, a fault characteristic frequency presence in the frequency spectrum is modulated, and it generates several fault harmonic frequencies. Thus, grouping these frequencies as a subset frequency band and examining its pattern will focus on this proposed method. The fault characteristic frequencies pattern is presented as,

(5) $$f^{f}=\left\{f_{1}^{f},f_{2}^{f},...,f_{k}^{f}\right\}$$

where $f_{k}^{f}$ is the *k*-th fault characteristic frequencies that induced into the current by the faulty conditions.

Misalignment is one of the most common faults present in a factory. It occurs in the transmission shaft between the motor and the driven system. The present misalignment in the industrial equipment may disrupt the transmission of power from the motor to the driven systems. It generates excessive vibration, and eventually, it leads to the driven system, coupling, and motor bearing failures. Thus, correcting the misalignment can prevent the industrial equipment from further failure.

Misalignment induces dynamic eccentricity in the motor. Dynamic eccentricity happens because the rotor center is mislocated from the center of rotation, and when the rotor rotates, the minor air-gap position rotates. Hence, the stator angle is affected by the air-gap in dynamic eccentricity as both the rotor and the minor air-gap position rotating [30]. The rotor position variation and oscillation in the length of the radial air-gap will induce the air-gap flux density variation into the stator current signal. Therefore, it generates two symmetrical side-bands around the IM operational frequency $f_{e}$ and its harmonic that appeared as the misalignment/unbalance fault signature,

(6) $$f_{miss}=f_{e}\pm m\times f_{r}$$

where m=1,3,5,⋯ and $f_{r}$ is the shaft rotational frequency. In this study, an angular misalignment experiment will be performed. The angular misalignment occurs when the centerline of the shaft connection is crooked and set an angle between the driven system and the motor. In particular, it damages both the motor and the driven system.

Besides misalignment as the common faulty condition, the other considerably faulty condition in which its fault signatures have never been isolated and mathematically expressed before will also be studied. A turbulent flow is considered a condition that frequently occurred in a pumping system. The turbulent flow occurs due to the pumping system running below the pump flow rate capacity. In some cases, the motor running at a speed below the minimum speed of the pump may cause the turbulent flow as well.

Similar to misalignment, the flow turbulence causes the centrifugal pump to vibrate. The vibrations travel through the shaft and couplings to the IM. These vibrations may interrupt the rotor rotation and induce air-gap fluctuations to the rotor-stator relationship. Thus, the flow turbulence may induce additional frequency disturbances to the stator current signal. There are no research reports found for MCSA in pump flow turbulence detection. Nonetheless, there is research done in centrifugal pump cavitation fault [31]. Since turbulence flow in along period leads to cavitation fault, the fault diagnosis approach presented in [31] will be referred. In contrast with angular misalignment, the turbulent flow has a no-fault signature modeled with MCSA. However, as the stator current signal is disturbed by the pump vibration, the presence of turbulent flow can be observed from the stator current signal, the same concept as misalignment. The presence of flow turbulence in a pump is not a fault. Nevertheless, it is a fault in the making.

Both misalignment and pump turbulent flow in a long period will damage both the driven equipment and the IM. The proposed FDI framework is expected to monitor the IM condition and the driven equipment condition. Thus, the proposed FDI framework employs the IM as a sensor to monitor the whole system of RM conditions and detect the fault presence.

## 3. Data-Driven Model Development

### 3.1. Subspace Identification

Consider the black-box identification, an IM in a quasi-steady-state condition can be represented by using a discrete-linear time-invariant state-space model. The discrete state-space describes the relationship between input, output, noise, and disturbance using a first-order difference equation system and auxiliary state vector $x_{k}$ as follows,

(7) $$\begin{array}{c} x_{k+1}=Ax_{k}+Bu_{k}+w_{k}\\ y_{k}=Cx_{k}+Du_{k}+v_{k} \end{array}$$

with

(8) $$E\left[\left(\begin{array}{c} w_{k}\\ v_{k} \end{array}\right)\left(\begin{array}{cc} {w_{k}}^{T} & {v_{k}}^{T} \end{array}\right)\right]=\left(\begin{array}{cc} Q & S\\ S^{T} & R \end{array}\right)\delta_{pq}\geq 0$$

where $y_{k}\in R^{l\times 1}$ and $u_{k}\in R^{m\times 1}$ are the measured output and input vectors, $x_{k}\in R^{n\times 1}$ is the state vectors, $w_{k}\in R^{n\times 1}$ and $v_{k}\in R^{l\times 1}$ are the unobservable disturbance and measurement noise vectors. $A\in R^{n\times n}$, $B\in R^{n\times m}$, $C\in R^{l\times n}$, and $D\in R^{l\times m}$ are the state, input-to-state, state-to-output, and feedthrough matrices. While $Q\in R^{n\times n}$, $S\in R^{n\times l}$, and $R\in R^{l\times l}$ the covariance matrices of Gaussian distributed zero-mean white noise $w_{k}$ and $v_{k}$. For the system to be able to be observed in the output $y_{k}$ and identified, the {A,C} is assumed to be observable. At the same time, the {$A,\left(\begin{array}{cc} B & Q^{1/2} \end{array}\right)$} is assumed to be controllable so that all the system modes can be excited by deterministic input $u_{k}$ and/or by stochastic input $w_{k}$. The input $u_{k}$ of the system is assumed to be persistently excited [21].

The SID algorithm interpretation and derivation lie on the state sequences and the extended observability matrix. The formulation of the state sequences and the observability matrix can be done by extending the state-space model in Equation (7) with the input and output data [32],

(9) $$Y_{f}=\Gamma_{i}X_{i}+H_{i}^{d}U_{f}+H_{i}^{s}M_{f}+N_{f},$$

where $\Gamma_{i}\in R^{li\times n}$ is the observability matrix defined as,

$$\Gamma_{i}\overset{\Delta }{=}{\left(\begin{array}{ccccc} C & CA & CA^{2} & \cdots & CA^{i-1} \end{array}\right)}^{T},$$

with the subscript “*d*” and “*s*” denote deterministic and stochastic meaning, the lower block triangular Toeplitz matrices $H_{i}^{d}\in R^{li\times mi}$ and $H_{i}^{s}\in R^{li\times mi}$ are defined as,

$$H_{i}^{d}\overset{\Delta }{=}\left(\begin{array}{ccccc} D & 0 & 0 & \cdots & 0\\ CB & D & 0 & \cdots & 0\\ CAB & CB & D & \cdots & 0\\ \vdots & \vdots & \vdots & \ddots & \vdots \\ CA^{i-2}B & CA^{i-3}B & CA^{i-4}B & \cdots & D \end{array}\right)$$

$$H_{i}^{s}\overset{\Delta }{=}\left(\begin{array}{ccccc} 0 & 0 & 0 & \cdots & 0\\ C & 0 & 0 & \cdots & 0\\ CA & C & 0 & \cdots & 0\\ \vdots & \vdots & \vdots & \ddots & \vdots \\ CA^{i-2} & CA^{i-3} & CA^{i-4} & \cdots & 0 \end{array}\right)$$

and the state sequences $X_{i}\in R^{n\times j}$ is defined as,

$$X_{i}\overset{\Delta }{=}\left(\begin{array}{ccccc} x_{i} & x_{i+1} & \cdots & x_{i+j-2} & x_{i+j-1} \end{array}\right)$$

In the SID algorithm, the input and output data have to be represented in block Hankel matrices. The length of block rows or horizon i is defined by the user and must be large enough compared to the maximum order *n*. The column of Hankel matrices *j* is set to be j=s−2i+1 with *s* is the length of available data, and it is assumed that j,s→∞. The input block Hankel matrix $U_{0|2i-1}\in R^{2mi\times j}$ is defined as,

(10) $$U_{0|2i-1}\overset{\Delta }{=}(\;\frac{\begin{array}{cccc} u_{0} & u_{1} & \cdots & u_{j-1}\\ u_{1} & u_{2} & \cdots & u_{j}\\ \vdots & \vdots & \ddots & \vdots \\ u_{i-1} & u_{i} & \cdots & u_{i+j-2} \end{array}}{\begin{array}{cccc} u_{i} & u_{i+1} & \cdots & u_{i+j-1}\\ u_{i+1} & u_{i+2} & \cdots & u_{i+j}\\ \vdots & \vdots & \ddots & \vdots \\ u_{2i-1} & u_{2i} & \cdots & u_{2i+j-2} \end{array}})\;$$

where

$$U_{0|2i-1}=(\;\frac{\begin{array}{c} U_{0|i-1} \end{array}}{\begin{array}{c} U_{i|2i-1} \end{array}}\;)=(\;\frac{\begin{array}{c} U_{p} \end{array}}{\begin{array}{c} U_{f} \end{array}}\;),\;U_{0|2i-1}=(\;\frac{\begin{array}{c} U_{0|i} \end{array}}{\begin{array}{c} U_{i+1|2i-1} \end{array}}\;)=(\;\frac{\begin{array}{c} {U_{p}}^{+} \end{array}}{\begin{array}{c} {U_{f}}^{-} \end{array}}\;)$$

Similarly, the output block Hankel matrix $Y_{0|2i-1}\in R^{2li\times j}$ can be defines as Equation (10).

In order to understand more regarding the system state sequences, several theories are explained and proven in [33]. It is denoted according to Theorem 11 in [33] that,

(11) $$Z_{i}\overset{\Delta }{=}\Gamma_{i}{\hat{X}}_{i}+H_{i}^{d}U_{f}=Y_{f}/\left(\begin{array}{c} U_{p}\\ Y_{p}\\ U_{f} \end{array}\right)$$

The projected matrix $Z_{i}$ is considered the optimal estimation of future output $Y_{f}$ based on the future input $U_{f}$, past input $U_{p}$, and past output $Y_{p}$ data without knowing the states of the system. By shifting the past and future border by one block rows, the projected matrix $Z_{i+1}$ can be found similarly as,

(12) $$Z_{i+1}\overset{\Delta }{=}\Gamma_{i-1}{\hat{X}}_{i+1}+H_{i-1}^{d}U_{f}^{-}=Y_{f}^{-}/\left(\begin{array}{c} U_{p}^{+}\\ Y_{p}^{+}\\ U_{f}^{-} \end{array}\right)$$

Hence, from Equations (11) and (12), it can be determined that,

(13) $${\hat{X}}_{i}=\Gamma_{i}^{†}\left[Z_{i}-H_{i}^{d}U_{f}\right]$$

(14) $${\hat{X}}_{i+1}=\Gamma_{i-1}^{†}\left[Z_{i+1}-H_{i-1}^{d}U_{f}^{-}\right],$$

where ${▪}^{†}$ denotes the Moore–Penrose pseudoinverse of the matrix ▪. First, to calculate the matrix $\Gamma_{i}$, the future output $Y_{f}$ row space is projected along with the future input $U_{f}$ row space into the joint of the row space between past input $U_{p}$ and past output $Y_{p}$. This projection is an oblique projection that yields to,

(15) $$O_{i}=Y_{f}{/}_{U_{f}}\left(\begin{array}{c} U_{p}\\ Y_{p} \end{array}\right).$$

In which an SVD method can be applied to decompose the matrix $O_{i}$ into,

(16) $$W_{1}O_{i}W_{2}=\left(\begin{array}{cc} U_{1} & U_{2} \end{array}\right)\left(\begin{array}{cc} \Sigma_{1} & 0\\ 0 & 0 \end{array}\right)\left(\begin{array}{c} V_{1}^{T}\\ V_{2}^{T} \end{array}\right)$$

where $W_{1}$ and $W_{2}$ are the user-defined weights that can be chosen as presented in [32]. The matrix $\Gamma_{i}$ then can be calculated by,

(17) $$\Gamma_{i}=U_{1}\Sigma_{1}^{1/2},$$

where the matrix $\Gamma_{i-1}$ can be obtained by not including the last *l* rows of the matrix $\Gamma_{i}$.

According to [33], it is explained and proven that the corresponding columns of ${\hat{X}}_{i}$ and ${\hat{X}}_{i+1}$ are the non-steady-state Kalman-filter state estimation at two consecutive time instants, therefore Equation (7) can be rewritten as,

(18) $$\left(\begin{array}{c} {\hat{X}}_{i+1}\\ Y_{i|i} \end{array}\right)=\left(\begin{array}{cc} A & B\\ C & D \end{array}\right)\left(\begin{array}{c} {\hat{X}}_{i}\\ U_{i|i} \end{array}\right)+\left(\begin{array}{c} \rho_{w}\\ \rho_{v} \end{array}\right)$$

where the $\rho_{w}$ and $\rho_{v}$ are the disturbance and noise matrices that their row spaces are orthogonal to the row spaces of $\left(\begin{array}{c} U_{p}\\ Y_{p} \end{array}\right)$, $U_{f}$, and ${\hat{X}}_{i}$. Substitute Equations (13) and (14) into Equation (18),

(19) $$\left(\begin{array}{c} \Gamma_{i-1}^{†}Z_{i+1}\\ Y_{i|i} \end{array}\right)=\left(\begin{array}{c} A\\ C \end{array}\right)\Gamma_{i}^{†}Z_{i}+KU_{f}+\left(\begin{array}{c} \rho_{w}\\ \rho_{v} \end{array}\right)$$

with

(20) $$K\overset{\Delta }{=}\left(\begin{array}{c} \left(\begin{array}{cc} B & \Gamma_{i-1}^{†}H_{i-1}^{d} \end{array}\right)\;-\;A\Gamma_{i}^{†}H_{i}^{d}\\ \left(\begin{array}{cc} D & 0 \end{array}\right)\;-\;C\Gamma_{i}^{†}H_{i}^{d} \end{array}\right)$$

The solution of Equation (19) can be found using the least-square method such that the matrices *A*, *C*, and K can be identified. According to (20), *B* and *D* appear to be linear with K, so it can be said that K(B,D) is a linear matrix function of *B* and *D*. Hence, a solution of K(B,D) can be found by using the least-square method to minimize,

(21) $$B,D=\arg\min_{B,D}∥\left(\begin{array}{c} \Gamma_{i-1}^{†}Z_{i+1}\\ Y_{i|i} \end{array}\right)-\left(\begin{array}{c} A\\ C \end{array}\right)\Gamma_{i}^{†}Z_{i}-K\left(B,D\right)U_{f}{∥}_{F}^{2}.$$

Suppose the matrices *A* and *C* are known by solving Equation (19) and the covariance matrix of the disturbances and the noises are known from Equation (8), the state covariance matrix can be found by solving a discrete Lyapunov equation,

(22) $$\Sigma^{s}=A\Sigma^{s}A+Q.$$

By defining the output covariance matrix, it can be found that,

(23) $$\Lambda_{0}=C\Sigma^{s}C+R.$$

Then, it can be defined that,

(24) $$G\overset{\Delta }{=}A\Sigma^{s}C^{T}+S$$

Finally, the Kalman filter gain can be calculated by,

(25) $$K=\left(G-APC^{T}\right){\left(\Lambda_{0}-CPC^{T}\right)}^{-1},$$

where *P* can be found by solving the algebraic Riccati equation,

(26) $$P=APA^{T}+\left(G-APC^{T}\right){\left(\Lambda_{0}-CPC^{T}\right)}^{-1}{\left(G-APC^{T}\right)}^{T}.$$

### 3.2. Residual Current Generation

Suppose an IM is described in the stationary frame, the stator current $i_{s\alpha }$ and $i_{s\beta }$, and the supply voltage $v_{s\alpha }$ and $v_{s\beta }$ are converted from the measurable three-phase reference ABC frame voltage $v_{a}$, $v_{b}$, $v_{c}$ and current $i_{a}$, $i_{b}$, $i_{c}$ by using Clarke’s Transform as follows,

(27) $$T_{\alpha \beta 0}=\frac{2}{3}\left(\begin{array}{ccc} \cos(\theta ) & \cos(\theta -\frac{2\pi }{3}) & \cos(\theta +\frac{2\pi }{3})\\ \sin(\theta ) & \sin(\theta +\frac{2\pi }{3}) & \sin(\theta -\frac{2\pi }{3})\\ \frac{1}{2} & \frac{1}{2} & \frac{1}{2} \end{array}\right),$$

where θ=0, and it applies to voltage and current as follows,

(28) $$\left(\begin{array}{c} v_{s\alpha }\\ v_{s\beta }\\ v_{s0} \end{array}\right)=T_{\alpha \beta 0}\left(\begin{array}{c} v_{a}\\ v_{b}\\ v_{c} \end{array}\right)$$

(29) $$\left(\begin{array}{c} i_{s\alpha }\\ i_{s\beta }\\ i_{s0} \end{array}\right)=T_{\alpha \beta 0}\left(\begin{array}{c} i_{a}\\ i_{b}\\ i_{c} \end{array}\right)$$

where $v_{s0}$ and $i_{s0}$ are equal to zero.

First, using the black-box identification presented as Algorithm 1, the IM discrete-state space model is obtained. Then by injecting the supply voltage $v_{s\alpha }$ and $v_{s\beta }$ into the identified state-space model, the estimated stator current ${\hat{i}}_{s\alpha }$ and ${\hat{i}}_{s\beta }$ are generated.
**Algorithm 1** IM state-space identification1:**input:** horizon *i*; voltage $v_{s\alpha }$ and $v_{s\beta }$; current $i_{s\alpha }$ and $i_{s\beta }$; and weight $W_{1}$ and $W_{2}$2:**output:** discrete state-space *A*, *B*, *C*, and *D*3:**procedure**SubspaceID($i,v_{s\alpha },v_{s\beta },i_{s\alpha },i_{s\beta },W_{1},W_{2}$)4:    **initialize** $U_{0|2i-1}$ and $Y_{0|2i-1}$                                                                ▹ Equation (10)5:    calculate $O_{i}$                                  ▹ Equation (15)6:    calculate $Z_{i}$ and $Z_{i+1}$                                                            ▹ Equations (11) and (12)7:    **do** $\left[U,\Sigma ,V\right]=svd\left(W_{1},O_{i},W_{2}\right)$8:    define n=49:    calculate $\Gamma_{i}$ and $\Gamma_{i-1}$                                                                             ▹ Equation (17)10:  **do** Least-Square problem to obtain *A*, *C*, and *K*                                  ▹ Equation (19)11:  **do** Least-Square problem to obtain *B* and *D*                                        ▹ Equation (21)12:**end procedure**

In order to execute the IM FDI, the residual current signal must be generated. The residual current signal is defined by subtracting the estimated stator current ${\hat{i}}_{s\alpha }$ and ${\hat{i}}_{s\beta }$ from the stator current $i_{s\alpha }$ and $i_{s\beta }$ then convert it back into the three-phase ABC frame by using inverse Clarke’s Transform as the following expression,

(30) $$\left(\begin{array}{c} r_{a}\\ r_{b}\\ r_{c} \end{array}\right)={\left(\begin{array}{c} T_{\alpha \beta 0} \end{array}\right)}^{-1}\left(\begin{array}{c} i_{s\alpha }-{\hat{i}}_{s\alpha }\\ i_{s\beta }-{\hat{i}}_{s\beta }\\ 0 \end{array}\right)$$

where $r_{a}$, $r_{b}$, and $r_{c}$ are the three-phase ABC frame residual current signal. The standard error estimate (SEE) is used to evaluate the fitness of the estimated current signal as follows,

(31) $$SEE=\sqrt{\frac{\sum_{k}{\left(r_{k}\right)}^{2}}{\sum_{k}{\left(i_{k}\right)}^{2}}},$$

where *r* is the residual signal and *i* is the measured current signal. Both are expressed in the three-phase ABC frame.

**Figure 2:**
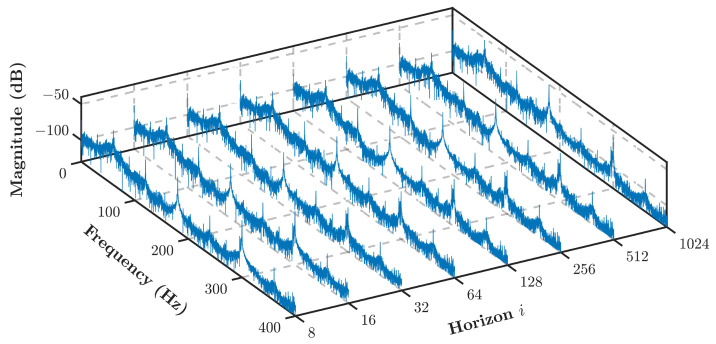
Various horizon *i* residual spectrum.

One of the requirements to implement Algorithm 1, a horizon *i* must be selected accordingly. Ref. [33] states that horizon *i* must be selected as i>n, with *n* as the state-space order. As the IM is assumed to be in a quasi-steady-state with a constant speed, the IM is described to be a fourth-order system (n=4) in this study. Several iterations to select an appropriate horizon *i* were performed. The selection categories are set to be computational time to perform the identification and the level of SEE. As it is presented in Table 1, the computational time varies a lot with tiny variations in the SEE level. Selecting i=32 will have almost 0.5% better SEE than i=16. Nevertheless, it has a trade-off of 0.1 seconds computational time. Selecting i=8 will increase the SEE level. Since the amount of data for the analysis is quite large, and considering computational time and acceptable SEE level, the horizon was selected to be i=16. Another reason is that the residual spectrum of these various horizons does not show much difference, as shown in Figure 2.

## 4. Fault Detection and Identification Algorithm

### 4.1. Residual Current Spectrum Threshold

**Figure 3:**
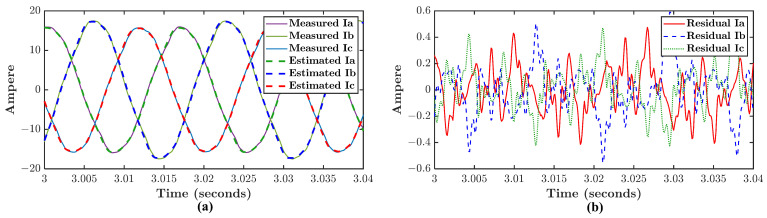
Three-phase signal of (**a**) measured and estimated current (**b**) residual current.

**Figure 4:**
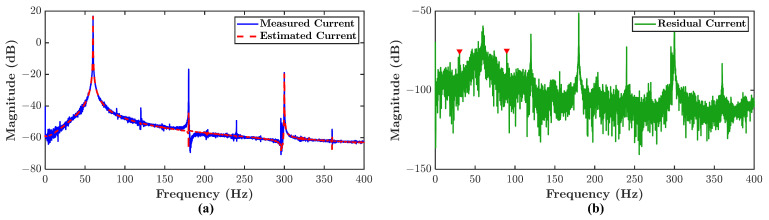
Power spectrum density of (**a**) measured and estimated current (**b**) residual current.

Consider measurement of voltage and current signals from an IM, where the current signal is described in Equation (1). By employing Algorithm 1, a model of this IM is identified from the measured voltage and current signals. The measured current is then subtracted by an estimated current generated from the identified model with the measured voltage as the input. The SEE is calculated via Equation (31) where the result is equal to 1.74%. Figure 3 shows the measured, estimated, and residual current of the IM in three-phase reference. The subtraction result is the residual current signal in which Equation (4) can be applied to obtain the residual spectrum. Figure 4 shows the measured, estimated, and residual current spectrums of a single-phase. It can be seen that the estimated spectrum is cleaner than the measured spectrum. At the same time, the residual spectrum contains the noise and harmonic residues as the result of subtraction. It is worth noting that sidebands can be noticed in the residual spectrum pointed by red arrows where it does not appear in the measured spectrum. The sidebands themselves are associated with misalignment signatures in Equation (6). There is likely an incipient misalignment fault presence in the IM. It appears that the potential advantage of the residual spectrum is more sensitive to fault signatures compare to the current spectrum.

An MCSA evaluation to determine an IM acceptable condition under a faulty condition has never been done because the fault signature magnitude can differ according to its operating condition. As the residual spectrum behaves more sensitively to the fault signatures, an evaluation must be performed to determine an acceptable condition of IM with fault signatures on its residual spectrum. In an actual industrial application, obtaining a dataset of an IM with perfect healthy conditions is impossible. Hence, this residual threshold can provide a solution in the residual spectrum evaluation, especially in an actual industrial application. The threshold is determined by a dataset of the relatively healthy residual spectrum. The so-called relatively healthy residual spectrum has a description as in Equation (3) but without faulty component or with faulty component but in acceptable low amplitude.

A dataset of *n* voltage and current pair from an IM is considered in healthy conditions and at the same loading is collected. By using Algorithm 1 and from this dataset, *n* IM models are identified. The *n* residual current signals are generated from the dataset by using these models. Through Equation (4), *n* single-phase residual signals are converted into *n* residual spectrums. Therefore, each residual frequency $f_{i}^{r}$ has a set of *n* magnitude values that is stored in matrix $R_{i}$.

(32) $$R_{i}=\left\{R_{1}\left(f_{i}^{r}\right),R_{2}\left(f_{i}^{r}\right),R_{3}\left(f_{i}^{r}\right),\cdots ,R_{n}\left(f_{i}^{r}\right)\right\},$$

where $R_{n}^{i}$ is the residual spectrum magnitude values of residual frequency $f_{i}^{r}$ at integer index *i*-th from the *n*-th measurement, and $R_{i}\in R^{1\times n}$ is the matrix where the dataset of *n* residual spectrum magnitude values is stored. The maximum magnitude value of frequency $f_{i}^{r}$ stored in $R_{i}$ can be defined as,

(33) $$R_{i_{max}}=\max\left(R_{i}\right),$$

while the mean and the standard deviation of these magnitude values can be calculated as,

(34) $${\bar{R}}_{i}=\frac{1}{n}\sum_{j=1}^{n}R_{j}\left(f_{i}^{r}\right)$$

(35) $$R_{i_{\sigma }}=\sqrt{\frac{1}{n-1}\sum_{j=1}^{n}\left(R_{j}\left(f_{i}^{r}\right)-{\bar{R}}_{i}\right)},$$

where ${\bar{R}}_{i}$ is the $f_{i}^{r}$ magnitude value mean and $R_{i_{\sigma }}$ is the $f_{i}^{r}$ magnitude value standard deviation. Based on these statistical evaluations, a threshold value for each frequency $f_{i}^{r}$ is interpreted as,

(36) $$R_{i_{TH}}=w_{1}\times \left({\bar{R}}_{i}+3\times R_{i_{\sigma }}\right)+w_{2}\times R_{i_{max}},$$

where $R_{i_{TH}}$ is the residual spectrum threshold at frequency $f_{i}^{r}$, and $w_{1}+w_{2}=1$ with $w_{1}\geq w_{2}$.

**Figure 5:**
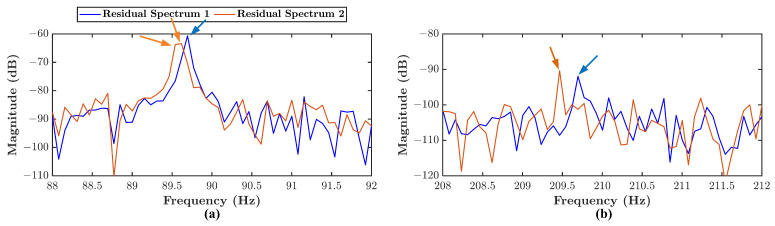
Fault signature peak of two different residual spectrum at (**a**) $f_{e}+1\times f_{r}$ (**b**) $f_{e}+5\times f_{r}$.

**Figure 6:**
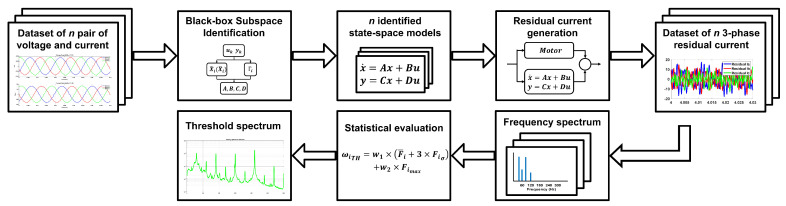
Proposed residual current spectrum threshold generator.

In an actual application, a frequency offset may occur. For example an IM is operated at $f_{e}=60$ Hz with rotor frequency at $f_{r}=28.5$ Hz. When the IM under faulty condition, according to Equation (6), the fault signature $f_{e}-1\times f_{r}$ appears in the residual spectrum at $f_{i}^{r}=31.5$ Hz. However, this signature will not always occur at 31.5 Hz, especially when *n* measurements are performed. In most data, the signature will occur at 31.5 Hz. In some data, it may occur at 31.2 Hz, and the other data, it may occur at 31.7 Hz. If one residual data has 130,000 data points and is obtained with a 10 kHz sampling rate, the maximum frequency for the residual spectrum is 5000 Hz with 65,000 index integers *i*. After a calculation, the integer of index *i* for the signature of $f_{i}^{r}=31.5$ Hz is obtained as i=410. At the same time, for the other measurements, the signature could be obtained at integer i=406 and i=412. This phenomenon is called the frequency offset. To obtain a clearer understanding of this phenomenon, Figure 5 is presented. This figure presents a comparison of two different residual spectrum data under the same angular misalignment condition in the same period of measurements. Figure 5a shows the offset of fault signature $f_{e}+1\times f_{r}$, and Figure 5b shows the the offset fault characteristic frequency of fault signature $f_{e}+5\times f_{r}$. Even, it can be seen that in Figure 5a for residual spectrum 2, the signature peak produces 2 integer index *i* with relatively similar magnitude. The offset distance of two different fault signatures is also different. The fault signature $f_{e}+5\times f_{r}$ has higher distance compare to fault signature $f_{e}+1\times f_{r}$. Figure 5 shows that the frequency offset can cause a bias in the significant peaks detection. Hence, the term $R_{i_{max}}$ in Equation (36) is used to compensate for the frequency magnitude that occurred in an offset frequency index. Meanwhile the term ${\bar{R}}_{i}+3\times R_{i_{\sigma }}$ is used to determine the threshold level of confidence, a threshold value can be chosen by tuning the weight parameters $w_{1}$ and $w_{2}$. The procedure to determine the healthy spectrum threshold can be seen in Figure 6.

The *n* identified models would be saved as the healthy model references to generate the estimated current signals. The *n* models are categorized by their normalized gain (current/voltage) and power vector. For example, observation data will select a model with similar gain and power factors to generate the residual signals, as shown in Figure 1. The model selection for observation data is accomplished by using the Euclidian distance calculation.

### 4.2. Binary Pattern of Fault Signature

According to Figure 1, the FDI process is started when the residual current spectrum of observation data is compared to the residual spectrum threshold that has been obtained from a set of trained data. As explained in Section 2, the main interest in the residual spectrum is the subset of frequencies whose magnitude is higher than the trained threshold. The residual spectrum and the threshold comparison is made index by index where the binary decision 1 and 0 is made. $Binary\_mat\in R^{K\times 1}$ is a matrix where the binary decision results are stored. *K* is the maximum integer of index *i*.

The Binary_mat generation is provided for two case studies of an IM in healthy and faulty conditions. This binary example is adapted from [34]. A residual spectrum is obtained from an IM in healthy conditions, and it is compared index by index with a threshold. Based on the 1 and 0 decision, the Binary_mat is generated as follows,

(37) $$Binary\_mat=\left[\begin{array}{ccccccccc} 0 & 1 & 0 & 0 & 0 & 0 & 0 & 1 & \cdots \end{array}\right]$$

While for a residual spectrum that is obtained from an IM in faulty condition, the Binary_mat is generated as follows,

(38) $$Binary\_mat=\left[\begin{array}{ccccccccc} 1 & 1 & 1 & 0 & 1 & 1 & 0 & 1 & \cdots \end{array}\right]$$

Based on those examples, the Binary_mat energy in Equation (38) is higher than in Equation (37) as the IM in faulty condition has more residual spectrum magnitudes higher than the threshold. Therefore, a detection metric can be determined through evaluation of the Binary_mat by normalizing its energy,

(39) $$f_{detect}=\frac{1}{K}\sum_{i=1}^{K}{Binary\_mat}_{i}$$

### 4.3. Fault Identification

Consider a dataset of *m* measurements. Take a single-phase residual spectrum from each measurement, and there are *m* residual spectrums available. Calculate the magnitude mean and standard deviation of each residual frequency $f_{i}^{r}$ as follows,

(40) $$\bar{R}\left(f_{i}^{r}\right)=\frac{1}{m}\sum_{j=1}^{m}R_{j}\left(f_{i}^{r}\right)$$

(41) $$R_{\sigma }\left(f_{i}^{r}\right)=\sqrt{\frac{1}{m-1}\sum_{j=1}^{m}\left(R_{j}\left(f_{i}^{r}\right)-\bar{R}\left(f_{i}^{r}\right)\right)}.$$

**Figure 7:**
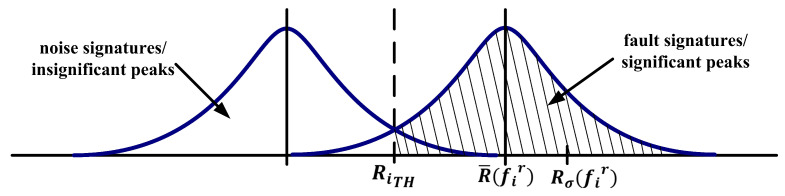
Probability distribution of fault and noise signatures.

Ideally, in the case of healthy IM, the Binary_mat in Equation (37) has only 0 s as its elements due to the residual spectrum must be less than the threshold. However, in the actual application, there are 1 s as its elements due to noise signatures/insignificant peaks occurred higher than the threshold. This condition occurs in faulty IM as well in Equation (38). Some 1 s that may not be associated with any fault signature. Thus, to determine whether 1 s belong to any specific fault signature or just belonged to random noise bands/insignificant peaks, an evaluation based on the probability distribution of fault and noise signatures is shown in Figure 7 must be done. The shaded area in Figure 7 represents the probability of fault signature/significant peaks, and the white areas represent the probability of noise signatures/insignificant peaks. The probability of fault or noise signatures can be calculated statistically through the *Q*-function as follows,

(42) $${Probability\_mat}_{i}=Q\left(\frac{R_{i_{TH}}-\bar{R}\left(f_{i}^{r}\right)}{R_{\sigma }\left(f_{i}^{r}\right)}\right),$$

where $Probability\_mat\in R^{K\times 1}$ contains the probability signatures of residual frequency $f_{i}^{r}$ for *m* measurements. Thus, the value of each Probability_mat element is ranged as $0\leq {Probability\_mat}_{i}\leq 1$. *Q*-function is chosen as it is proven to be suitable for fault detection [34,35]. In this study, it is employed further for fault identification.

Theoretically, the 1s associated with any specific fault signature will always occur on the specific frequency $f_{i}^{r}$ for *m* times. Hence, the Probability_mat value at that specific frequency $f_{i}^{r}$ is equal to 1. However, if there is a frequency offset, the Probability_mat value at that specific frequency $f_{i}^{r}$ can be less than 1, but the summation probability of nearby frequencies must be close to 1. At the same time, the 1s associated with the noise bands/insignificant peaks will occur on the specific frequency $f_{i}^{r}$ randomly with probability distribution shows in Figure 7.

Suppose $H_{0}$ is the null hypothesis states that a peak higher than the threshold is a fault signature/significant peak, and $H_{1}$ is the alternative hypothesis states that a peak higher than the threshold is a noise signature/insignificant peak. Because of the frequency offset possibility, the determination of 1 s that associated with any specific fault can be done through integer index *i* sliding window summation as follows,

(43) $$\left\{\begin{array}{cc} H_{0}, & \mathrm{if}\;\sum_{j=-M}^{M}{Probability\_mat}_{i+j}\geq p_{TH}\\ H_{1}, & \mathrm{if}\;\sum_{j=-M}^{M}{Probability\_mat}_{i+j}<p_{TH}, \end{array}\right.$$

where *M* is the integer index *i* sliding window that determines the number of previous and next integer indexes, and $p_{TH}$ is a user-defined threshold that may compensate the rate of fault identification. Through Equation (43), the frequency $f_{i}^{r}$ in any residual spectrum that associated with any certain fault signature can be identified as $f_{k}^{f}$ in Equation (5) by,

(44) $$\left\{\begin{array}{cc} f_{k}^{f}=f_{i}^{r}, & \mathrm{if}\;H_{0}\;\mathrm{is}\;\mathrm{true}\\ f_{k}^{f}\neq f_{i}^{r}, & \mathrm{if}\;H_{1}\;\mathrm{is}\;\mathrm{true} \end{array}\right.$$

where $f_{k}^{f}$ is the *k*-th fault signature. As it is known that between one residual spectrum and another residual spectrum, the fault characteristic frequency may differ because of the frequency offset. Therefore, the $f_{k}^{f}$ is a frequency band collection where the fault signatures most probably occur. Then, the Binary_mat is tested against the fault frequency bands $f_{k}^{f}$. If the 1 s in Binary_mat lay inside the fault frequency bands $f_{k}^{f}$, they are valid as the fault signatures. Nevertheless, if the 1 s lies outside the fault frequency bands $f_{k}^{f}$, they are invalid fault signatures and set as 0 s. Once the Binary_mat is updated, Equation (39) can be used for more robust detection metric.

Finally, for each residual spectrum, the value of the magnitude of fault frequencies can be found according to each residual spectrum ${Binary\_mat}_{i}$ wherein index *i* contains 1. These magnitude values are stored as $R_{k}\left(f^{f}\right)$. Then, the magnitude value of *p* identified fault frequencies in the residual spectrum can be used to determine the severity of the fault as follows,

(45) $$f_{severity}=\sum_{k=1}^{p}R_{k}\left(f^{f}\right).$$

The procedure is presented as Algorithm 2.
**Algorithm 2** Fault Detection and Identification1:**for** j=1,2,3,…,m**do**                 ▹ generate the Binary_mat2:    **for** i=1,2,3,…,K **do**3:        **if** $R_{j}\left(f_{i}^{r}\right)>R_{i_{TH}}$ **then**4:           $Binary\_mat_{i,j}=1$5:        **else**6:           $Binary\_mat_{i,j}=0$7:        **end if**8:     **end for**9:  **end for**10:**for**i=1,2,3,…,K**do**              ▹ generate the Probability_mat11:    calculate $Probability\_mat_{i}$                      ▹ Equation (42)12: **end for**13:**initialize** *M* as index sliding windows         ▹ identify the fault frequency bands $f_{k}^{f}$14:**for** i=1,2,3,…,K **do**15:    **if** $\sum_{j=-M}^{M}{Probability\_mat}_{i+j}\geq p_{TH}$ **then**              ▹ Equation (43)16:        $H_{0}$ is true, set the fault frequency band as $f_{k}^{f}=f_{i}^{r}$             ▹ Equation (44)17:    **end if**18:**end for**19:**for**j=1,2,3,…,m **do**         ▹ update the Binary_mat according to the $f_{k}^{f}$20:    **for** i=1,2,3,…,K **do**21:        **if** $Binary\_mat_{i,j}=1$ and $any(index\left(f_{k}^{f}\right)=j)=1$ **then**22:             $Binary\_mat_{i,j}=1$23:        **else**24:           $Binary\_mat_{i,j}=0$25:        **end if**26:    **end for**27:**end for**28:calculate $f_{detect}$                               ▹ Equation (39)29:calculate $f_{severity}$                            ▹ Equation (45)

## 5. Experimental Setup

**Figure 8:**
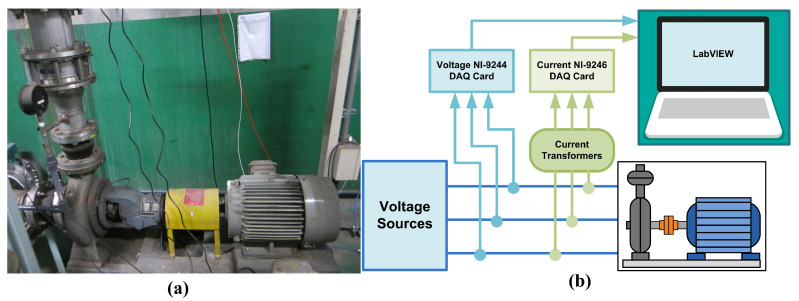
Experimental setup: (**a**) wastewater centrifugal pump-induction motor platform (**b**) data acquisition schematic.

This study is conducted on a wastewater centrifugal pump driven by an IM with a flexible coupling transmission, as shown in Figure 8a. The IM has the following rated parameters shown in Table 2. The experimental setup schematic representation is shown in Figure 8b. Three 25:5A class 1 current transformers (CT) with ±1% error tolerance are installed to measure the three-phase current signal. The class 1 CTs are used to obtain high SNR in the measured signal. The 5A output of the current transformers is acquired by using the NI-9246 DAQ card. The three-phase voltage signal is acquired by using the NI-9244 DAQ card. A 10 kHz sampling frequency samples both signals. The DAQ user interface is developed in the NI LabVIEW software environment. In addition, vibration measurements are performed as well in the turbulent flow experiments. An accelerometer transducer is installed in the radial position on the pump side. The measurement employs a 10 kHz NI DAQ card. For each aforementioned input opening, five vibration data are measured. Measuring the vibration data in an actual industrial facility is quite a challenge because the transducer cannot be installed in the optimum location, and the vibration noise is high.

Two types of experiments are done such as misalignment and turbulent flow. The misalignment is a common fault in the industry, and its fault signatures can be obtained using Equation (6). Moreover, the turbulent flow in a pump is a phenomenon that must be avoided, especially in a pumping system, as it may lead to the cavitation fault if it is allowed to happen for quite an extended period. In contrast to misalignment, there is no fault signature of turbulent flow presented in a mathematical expression. Thus, turbulent flow detection and identification rely on the presence of a frequency pattern in which its magnitude is above the residual threshold.

Three types of angular misalignment faults are executed by adding a washer for each IM back-side foot. Type 1: 0.2 mm thickness, type 2: 0.5 mm thickness, and type 3: 1.5 mm thickness. Meanwhile, the turbulent flow is induced in the pump by abruptly changing the pump input valve opening with 100%, 80%, 60%, and 50% openings. During the turbulent flow experiments, the pump output valve opening is set to be 50%. Closing the pump input valve is expected to reduce the volume of water entering the pump. Hence, reduce the pressure difference and induce both air and water in the pump and thus generate a turbulent flow in the pump.

Table 3 shows the parameters used for the FDI algorithm presented in Section 4,

## 6. Results and Discussions

### 6.1. Angular Misalignment

A set of 30 measurements is collected for each condition in the misalignment fault experiment, including healthy and three conditions of angular misalignment fault. The residual threshold is obtained from a set combination of 60 measurements on six different dates when the IM is running in a considerably healthy condition. It is common that fault detection is performed on the time-domain residual data [8,13,17]. However, time-domain residual data evaluation for fault detection will determine the success of the fault detection if the fault alters the output of the system, as an example, the current signal in our study. Hence, the difference can be seen from the time-domain residual data.

**Figure 9:**
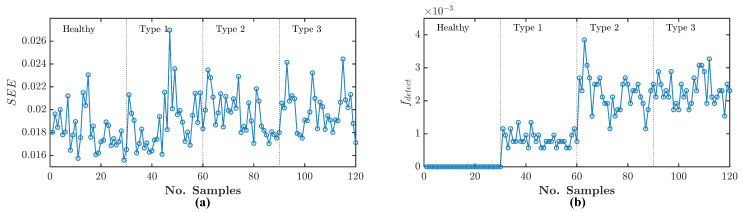
Angular misalignment experiment analysis using Equations (31) and (39): (**a**) SEE data, (**b**) $f_{detect}$ data.

Figure 9a shows the SEE time-domain data of the healthy and three angular misalignment conditions. At the same time, Figure 9b shows the $f_{detect}$ frequency-domain data. The difference between the healthy and misalignment conditions is hardly distinguishable in the SEE results because the misalignment fault signature magnitudes are too small to alter the output current signal. Thus, there is not much difference between healthy and misalignment residual SEE. However, it is distinguishable in the $f_{detect}$ data. The $f_{detect}$ shows distinguishable difference because it is calculated from the updated Binary_mat with respect to Equation (44). Hence, all of the 1 s in Binary_mat that is related to noise signatures are set to be 0 s—leaving the only 1 s that are related to fault signature. It can be seen from the healthy condition that the $f_{detect}$ is equal to 0 for all the samples because there is no detected fault signature leaving the Binary_mat with 0 s only. At the same time, for misalignment conditions $f_{detect}$ shows non-zero values. $f_{detect}$ surpasses SEE because its evaluation depends only on the presence of fault signatures in the frequency-domain.

**Figure 10:**
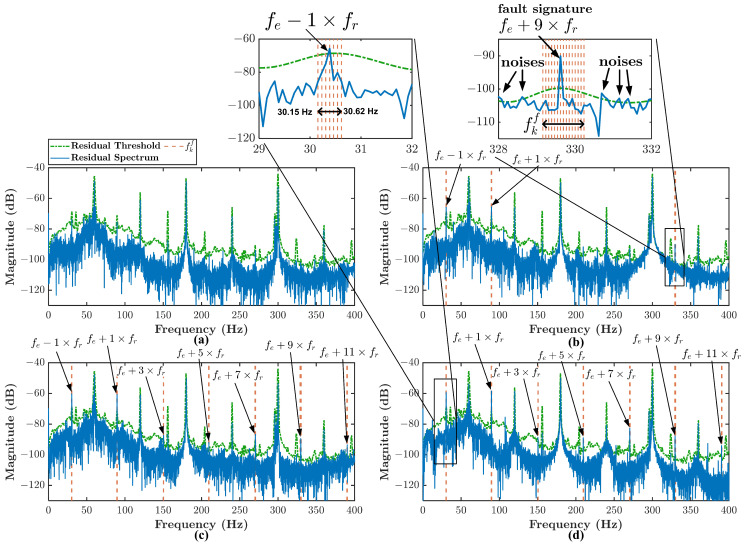
Angular misalignment residual spectrum: (**a**) healthy condition, (**b**) type 1 condition, (**c**) type 2 condition, (**d**) type 3 condition.

The residual spectrum can further be analyzed to identify the fault signatures. Figure 10 shows the residual spectrum example of healthy and three types of misalignment conditions. For the sake of clear figure explanation, there are two zoomed-in frequency bands in Figure 10b,d. The frequency band in orange color is the fault band $f_{k}^{f}$ obtained from Equation (44). There is no identified fault band $f_{k}^{f}$ in Figure 10a. The less severe misalignment condition such as type 1, the less fault band $f_{k}^{f}$ number identified. From the zoomed-in frequency band, it can be seen that the fault signature can be identified as a peak higher than the residual threshold and lies inside the fault band $f_{k}^{f}$. The peak is the fault signature described in Equation (6). Several peaks higher than the residual threshold are considered noise because they lay outside the fault band $f_{k}^{f}$. For type 2 and type 3 misalignment, the fault band $f_{k}^{f}$ number is higher. Hence, the identified fault signature number is also higher. The difference between type 2 and type 3 misalignment lies in the energy of those fault signatures.

Sample of identified fault signatures from 5 residual spectrums for type 3 misalignment is presented in Table 4. Fault signature column corresponds to Equation (6). The frequency column corresponds to the theoretical characteristic frequency where the fault signature from Equation (6) may occur. $f_{i}^{r}$ columns correspond to which residual frequency with integer *i* is identified as the fault characteristic frequency according to the related fault signature.

Based on Table 4, it can be seen that at fault signature $f_{e}-1\times f_{r}$, there are three frequencies related to this signature because of the frequency offset phenomenon. They are identified as 30.31 Hz, 30.39 Hz, and 30.46 Hz. The fault signature $f_{e}-1\times f_{r}$ suppose to appear at 30.35 Hz, but the frequency is offset in three different frequencies. Hence, through Equations (42)–(44), the fault frequency band $f_{k}^{f}$ for fault signature $f_{e}-1\times f_{r}$ is identified between 30.15 to 30.62 Hz. This fault band can be seen in zoomed-in fault signature $f_{e}-1\times f_{r}$ in Figure 10d. Any fault characteristic frequency that occurs inside this band and is higher than the residual threshold will be considered the fault signature $f_{e}-1\times f_{r}$. A similar explanation applies to the other fault signatures presented in Table 4.

Finding the fault frequency band $f_{k}^{f}$ is proven to be effective to determine the fault characteristic frequency subjected to the frequency offset phenomenon and filters-out any insignificant peaks/noise signature for robust Binary_mat and $f_{detect}$. The frequency offset phenomenon occurs due to the sampling frequency, data duration, and the number of data points acquired. This method can be used to achieve automated fault detection and identification without prior knowing the fault signatures.

**Figure 11:**
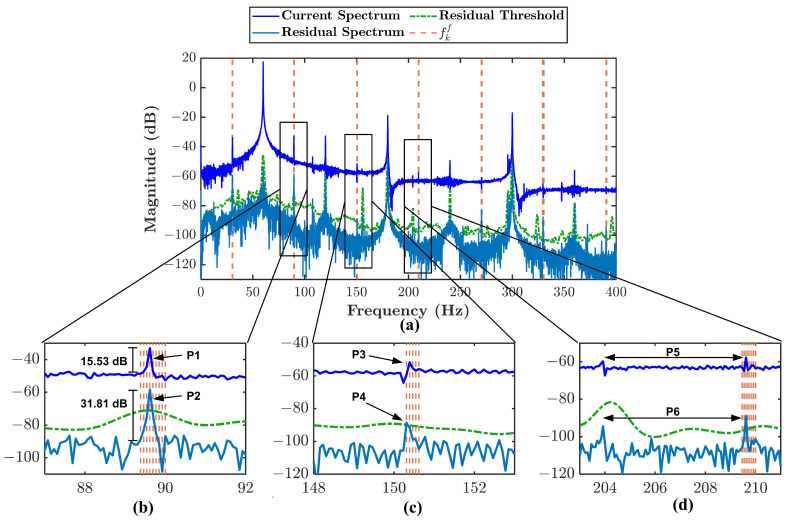
Comparison between current spectrum and residual current spectrum together with residual threshold and the fault frequency bands $f_{k}^{f}$: (**a**) type 3 misalignment condition, (**b**) zoomed-in frequency band around $f_{e}+1\times f_{r}$, (**c**) zoomed-in frequency band around $f_{e}+3\times f_{r}$, (**d**) zoomed-in frequency band around $f_{e}+5\times f_{r}$.

A comparison between MFDI is compared to MCSA to evaluate the effectiveness of the proposed method. Figure 11 shows the comparison between MFDI represented by residual spectrum and MCSA represented by current spectrum. Only type 3 misalignment condition is chosen because the other types have a similar comparison. Figure 11b–d are used to explain the difference between current spectrum and residual spectrum.

The first comparison is made based on Figure 11b. P1 points to $f_{e}+1\times f_{r}$ signature in the current spectrum, P2 points to the same signature in the residual spectrum. It can be seen that the energy of the signature is enhanced twice higher in the residual spectrum. It happens because of the removal of dominant harmonics energy in the spectrum by subtracting the current signal with the estimated current signal. Therefore, it makes the fault signature more evident in the residual spectrum.

Second comparison is presented in Figure 11c. P3 points to $f_{e}+3\times f_{r}$ signature in the current spectrum, P4 points to the same signature in the residual spectrum. The magnitude of signature pointed by P3 is relatively small to be considered as the fault signature. However, the signature can be considered the fault signature because of the residual threshold and the fault band $f_{k}^{f}$. It makes the residual spectrum more sensitive in fault signature identification.

Another advantage of residual spectrum is presented in Figure 11d. P5 points to identical signatures in the current spectrum, P6 points to the same signature in the residual spectrum. It is difficult to determine whether the first peak will also be considered a fault signature in the current spectrum. It is also difficult to evaluate whether it contributes to the fault severity. Nevertheless, using the proposed method, the first peak is not considered a fault signature since it simply lies outside the fault band and is lower than the threshold. Then, there is no contribution for that peak in fault severity. It proves that the proposed method can avoid false signature identification.

**Figure 12:**
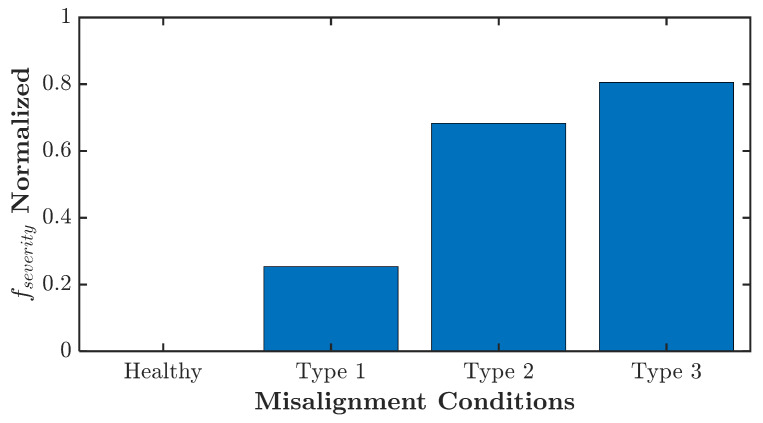
Angular misalignment experiment normalized fault severity level.

Figure 12 shows the results of normalized fault severity evaluation. The fault severity is evaluated by using Equation (45), and technically, it is a summation of the energy of the identified fault frequencies. A mean value is calculated from 30 data in each condition. Since there is no fault signature identified in the healthy condition, no energy can be summed. Therefore, it indicates zero levels of fault severity. The normalized fault severity is highly dependent on the user-defined level. It depends on how the user will allow the IM to run in such a faulty condition. It is possible to allow the IM to run in more severe misalignment conditions than type 3. Thus, the maximum normalized fault severity level will be higher. However, the idea in Figure 12 is to present the severity evaluation based on the identified fault frequency energy. It provides a quantified severity evaluation.

### 6.2. Turbulent Flow

The flow turbulence experiment is executed by undertaking the abrupt changes in the pump input valve opening. Three types of abrupt change of pump input valve opening and one standard 100% opening are performed, and 30 measurements for each condition are done. The experiment is performed by letting the pump operated with 100% input valve opening, and then the valve is closed to 80% opening, to 60% opening, to 50% opening, and return to standard 100% opening as the beginning. According to vibration reports in [36], the flow turbulence indicates by the high-frequency bands.

**Figure 13:**
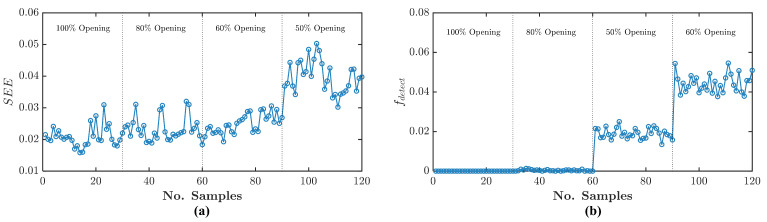
Turbulent flow experiment analysis using Equations (31) and (39): (**a**) SEE data, (**b**) $f_{detect}$ data.

Similar to the misalignment condition, the turbulent flow condition can be observed from the SEE level and the $f_{detect}$. Figure 13a,b shows the turbulent flow experiment SEE level and $f_{detect}$ respectively. The first three different input openings cannot be distinguished clearly by using the SEE level. However, for 50% opening, it shows higher and distinguishable SEE. In contrast to the SEE level, the two last openings can be distinguished clearly. According to this result, it can be confirmed that the flow turbulence appeared when there is an abrupt change in the input valve openings at 60% and 50%. However, it can be observed more in detail using the residual spectrum of these different input valve openings.

**Figure 14:**
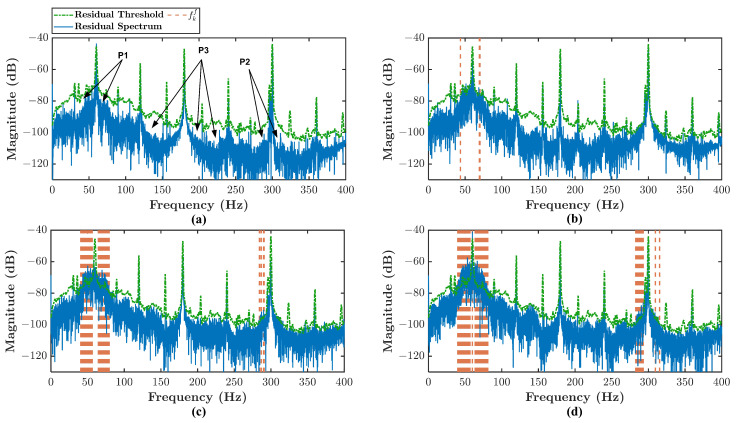
Turbulent flow residual spectrum: (**a**) 100% input valve opening (**b**) 80% input valve opening (**c**) 60% input valve opening (**d**) 50% input valve opening.

The example of residual spectrum for different input valve opening can be seen in Figure 14. In the turbulent flow analysis, the same residual threshold used in misalignment analysis is employed. P1, P2, and P3 point to frequency bands around 1×, 3×, and 5× harmonics respectively as shown in Figure 14a. Figure 14a shows the residual spectrum of 100% input valve opening. Hence, as is expected, no signature is detected. Once, the input valve is abruptly closed about 20% sudden rising of residual spectrum magnitude is noticeable in around 1× and 5× harmonics shown in Figure 14b. There are small fault bands $f_{k}^{f}$ identified around 1× harmonic. However, the fault band is getting larger when the input valve is abruptly closed about 40% and 50% as shown in Figure 14c,d. Even in the 60% input valve opening, an additional fault band identified close to 5× harmonic where it is getting more prominent in the 50% opening. The sudden rising of residual spectrum magnitude for 60% and 50% openings can be noticed around 1×, 3×, and 5× harmonics. However, it is only around 1× and 5× harmonics where they rise higher than the threshold.

Based on the residual spectrum analysis, the turbulence flow signatures occur as a frequency band rather than a single peak or a side-band signature as in the misalignment condition. Thus, it can be said that no flow turbulence occurred in 100% and 80% input valve openings. The flow turbulence occurs in 60% and 50% input valve openings. It is because the small input valve opening blocks and interrupts the fluid flow and reduces the pressure, which resulted in a turbulent flow. The turbulent flow can indicate input valve operation issues, whether manually or automatically operated. An issue in the input valve operation also leads the pump to run below its capacity.

**Figure 15:**
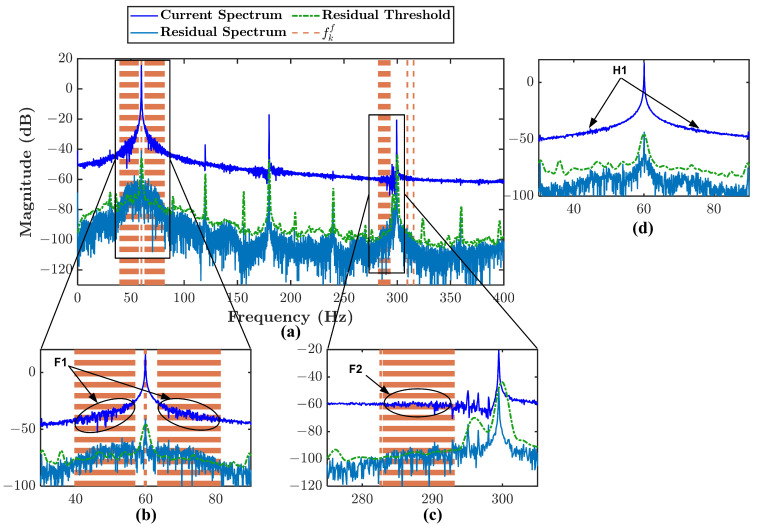
Comparison between current spectrum and residual current spectrum together with residual threshold and the fault frequency bands $f_{k}^{f}$: (**a**) 50% input valve opening, (**b**) zoomed-in frequency band around 1× harmonic, (**c**) zoomed-in frequency band around 3× harmonic, (**d**) zoomed-in frequency band around 1× harmonic of 100% input valve opening (this is presented for additional help in the comparison between turbulence and no turbulence spectrum).

The proposed MFDI and MCSA are also compared for turbulent flow detection through current and residual spectrum comparison. The comparison is presented only for 50% input valve opening. Figure 15 shows the comparison between the current and residual spectrum.

The first comparison is presented to examine the pattern identified around 1× harmonic and it is presented in Figure 15b. F1 points to the mirror wrinkle patterns that lay inside the fault band $f_{k}^{f}$. At the same time, the patterns are not visible in 100% opening as pointed by H1 in Figure 15d. According to research presented in [31], a cavitation fault can be detected through the current spectrum by observing a mirror frequency band around 1× harmonic. It similar pattern as P1 points it, but with smaller magnitudes. It is essential to notice that if turbulent flow lasts for a more extended period, it eventually leads to cavitation fault. It can be the reason that the flow turbulence shows a similar signature pattern as it is reported in [31]. As in the residual spectrum, all the dominant harmonic energy is removed, the pattern can be seen more obvious. In the second comparison, a similar pattern can be observed close to the 5× harmonic in Figure 15c as pointed by F2. However, it is not as visible as the residual spectrum indicates.

**Figure 16:**
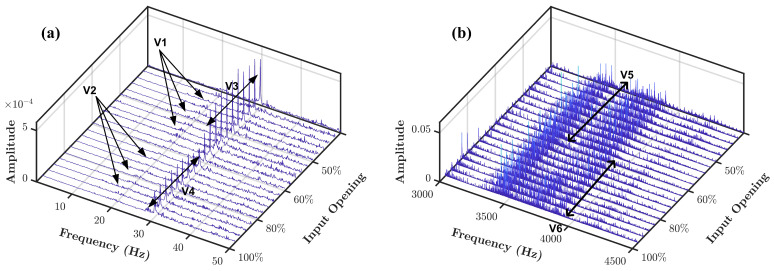
Radial vibration of centrifugal pump in four different input valve openings: (**a**) frequency band 0 to 50 Hz, (**b**) frequency band 3000 to 4500 Hz.

In order to complete the comparison analysis, a radial vibration spectrum waterfall of the experimental pump is presented in Figure 16. According to [36], the flow turbulence signature can be observed in the vibration spectrum as a frequency band pattern below vibration 1× harmonic and as a higher frequency band pattern. A similar pattern also visible in the vibration spectrum of a pump suffers from cavitation fault as reported in [31].

The pattern of the turbulence flow can be seen in the vibration spectrum for 60% and 50% openings as pointed by V1.However, the turbulence pattern is not visible for 100% and 80% openings, as pointed by V2.The magnitude of the vibration 1× harmonic pointed by V3 and V4, respectively, is a distinguishable difference of turbulence and no turbulence condition. At the higher frequency band, the magnitude difference is pointed by V5 and V6 for turbulence and no turbulence condition, respectively. Due to the high vibration noise, the difference cannot be seen clearly.

The turbulent flow is not a cavitation fault. Nevertheless, if the condition is kept for a more extended period, it develops into a cavitation fault. Detecting the turbulent flow phenomenon helps avoid further issues and keeps the pump running in its optimal condition and capacity. Based on the presented comparison, the residual current spectrum provides better sensitivity in turbulent flow signatures identification.

**Figure 17:**
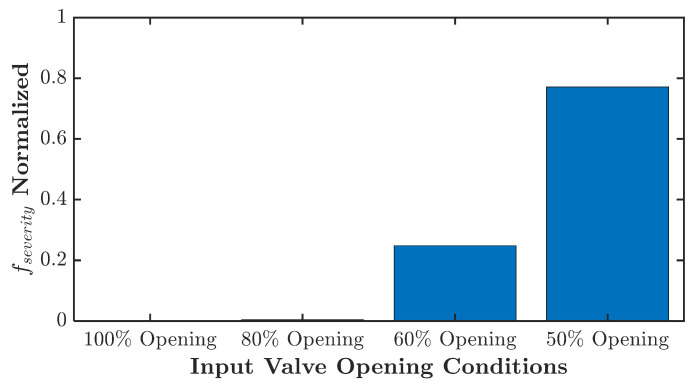
Turbulent flow experiment normalized fault severity level.

A quantified severity evaluation is presented in Figure 17. It shows that the turbulence severity for 60% opening is much lower than the 50% openings. At the same time, there is zero level fault severity for 100% openings since there is no energy to be summed. This fault severity quantification can help to determine further corrective action for the faulty condition.

## 7. Conclusions and Future Works

### 7.1. Conclusions

A novel MFDI framework to improve the IM-driven RM FDI is proposed in this study. The framework requires a discrete linear IM state-space model obtained using a data-driven SID algorithm assuming that the IM-driven system runs in quasi-steady-state condition. The MFDI utilizes the residual spectrum, residual threshold, and fault frequency bands. The combination of residual threshold and fault frequency band provides robust identification of fault signatures. In an undertaken comparison with MCSA, the proposed MFDI provides better sensitivity and identification results. The identified fault signature energy can also be used to determine fault severity level for further maintenance decisions. The experiment in an actual industrial wastewater facility also proves that the proposed MFDI framework provides a promising solution in developing an industrial-level FDI framework. An insight into an actual industrial application is that the difficulty in obtaining a perfect IM in healthy conditions can be overcome by adjusting the residual spectrum threshold according to the available set of IM data considered in healthy conditions.

### 7.2. Future Works

This presented study is performed in a centrifugal pumping system, assuming that the system runs in a quasi-steady-state condition. Further study will be performed in a system that runs in different operating frequencies and loading. In this scenario, a set of state-space models and residual thresholds must be identified and generated for each different operating condition. A cluster of state-space models and residual thresholds can be created based on the operating frequency and loading. A similar method to gain-scheduling will be developed for the appropriate selection of the state-space model and the residual threshold in the generated clusters.

## Figures and Tables

**Table 1:** Computational time and SEE level comparison for various horizon *i*.

Horizon *i*	Time (s)	SEE (%)
8	0.05	1.75
16	0.09	1.59
32	0.19	1.12
64	0.51	1.41
128	1.20	1.41
256	3.11	1.49
512	15.01	1.45
1028	90.97	1.55

**Table 2:** Wastewater pump induction motor specification.

Parameters	Value	Unit
Phase	3	-
Poles	4	-
Power	17	kW
Frequency	60	Hz
Rated Current	17	A
Rated Voltage	480	V
Rated Speed	1760	rpm

**Table 3:** FDI algorithm parameters.

Parameters	Value	Unit
$w_{1}$	0.6	-
$w_{2}$	0.4	-
*M* (index)	3	indices
$p_{TH}$	0.5	-

**Table 4:** Type 3 angular misalignment identified fault signatures example of 5 residual spectrum data.

		Identified Fault Frequencies									
Fault Signature	Frequency (Hz)	1		2		3		4		5	
		$f_{i}^{r}$	i	$f_{i}^{r}$	i	$f_{i}^{r}$	i	$f_{i}^{r}$	i	$f_{i}^{r}$	i
**$f_{e}-1\times f_{r}$**	30.35	30.39	396	30.46	397	30.31	395	30.31	395	30.39	396
**$f_{e}+1\times f_{r}$**	89.65	89.69	1167	89.77	1168	89.62	1166	89.54	1165	89.62	1166
**$f_{e}+3\times f_{r}$**	148.95	150.39	1956	150.62	1959	150.31	1955	150.23	1954	150.31	1955
**$f_{e}+5\times f_{r}$**	208.25	209.7	2727	210	2731	209.54	2725	209.46	2724	209.54	2725
**$f_{e}+7\times f_{r}$**	267.55	270.47	3517	270.85	3522	270.23	3514	270.16	3513	270.23	3514
**$f_{e}+9\times f_{r}$**	326.85	329.78	4288	330.24	4294	329.47	4284	329.39	4283	329.47	4284
**$f_{e}+11\times f_{r}$**	386.15	390.47	5077	391.08	5085	390.16	5073	390.01	5071	390.16	5073

## Data Availability

The data presented in this study are available upon request to the corresponding author.

## Abbreviations

The following abbreviations are used in this manuscript:

CT	Current Transformer
DAQ	Data Acquisition
DFT	Discrete Fourier Transform
FDI	Fault Detection and Identification
FFT	Fast Fourier Transform
IM	Induction Motor
MCSA	Motor Current Signature Analysis
MFDI	Model-based Fault Detection and Identification
PDF	Probability Density Function
RM	Rotating Machinery
SEE	Standard Error Estimate
SID	Subspace Identification
SNR	Signal-to-Noise Ratio
SVD	Singular Value Decomposition
